# Preliminary evaluation of safety and migration of immune activated mesenchymal stromal cells administered by subconjunctival injection for equine recurrent uveitis

**DOI:** 10.3389/fvets.2023.1293199

**Published:** 2023-12-14

**Authors:** Jennifer M. Cassano, Brian C. Leonard, Bianca C. Martins, Natalia Vapniarsky, Joshua T. Morgan, Steven W. Dow, Kathryn L. Wotman, Lynn M. Pezzanite

**Affiliations:** ^1^Veterinary Institute for Regenerative Cures, School of Veterinary Medicine, University of California, Davis, Davis, CA, United States; ^2^Department of Medicine and Epidemiology, School of Veterinary Medicine, University of California, Davis, Davis, CA, United States; ^3^Department of Surgical and Radiological Sciences, School of Veterinary Medicine, University of California, Davis, Davis, CA, United States; ^4^Department of Ophthalmology & Vision Science, School of Medicine, University of California, Davis, Davis, CA, United States; ^5^Department of Pathology, Microbiology, and Immunology, School of Veterinary Medicine, University of California, Davis, Davis, CA, United States; ^6^Department of Bioengineering, University of California, Riverside, Riverside, CA, United States; ^7^Department of Clinical Sciences, College of Veterinary Medicine and Biomedical Sciences, Colorado State University, Fort Collins, CO, United States; ^8^Department of Microbiology, Immunology and Pathology, College of Veterinary Medicine and Biomedical Sciences, Colorado State University, Fort Collins, CO, United States

**Keywords:** regenerative medicine, mesenchymal stromal (stem) cell (MSC), equine, subconjunctival therapy, equine recurrent uveitis (ERU), immunomodulatory therapy

## Abstract

**Introduction:**

Equine recurrent uveitis (ERU), an immune mediated disease characterized by repeated episodes of intra-ocular inflammation, affects 25% of horses in the USA and is the most common cause of glaucoma, cataracts, and blindness. Mesenchymal stromal cells (MSCs) have immunomodulatory properties, which are upregulated by preconditioning with toll-like receptor agonists. The objective was to evaluate safety and migration of TLR-3 agonist polyinosinic, polycytidylic acid (pIC)-activated MSCs injected subconjunctivally in healthy horses prior to clinical application in horses with ERU. We hypothesized that activated allogeneic MSCs injected subconjunctivally would not induce ocular or systemic inflammation and would remain in the conjunctiva for >14 days.

**Methods:**

Bulbar subconjunctiva of two horses was injected with 10 × 10^6^ pIC-activated (10 μg/mL, 2 h) GFP-labeled MSCs from one donor three times at two-week intervals. Vehicle (saline) control was injected in the contralateral conjunctiva. Horses received physical and ophthalmic exams [slit lamp biomicroscopy, rebound tonometry, fundic examination, and semiquantitative preclinical ocular toxicology scoring (SPOTS)] every 1–3 days. Systemic inflammation was assessed via CBC, fibrinogen, and serum amyloid A (SAA). Horses were euthanized 14 days following final injection. Full necropsy and histopathology were performed to examine ocular tissues and 36 systemic organs for MSC presence via IVIS Spectrum. Anti-GFP immunohistochemistry was performed on ocular tissues.

**Results:**

No change in physical examinations was noted. Bloodwork revealed fibrinogen 100-300 mg/dL (ref 100–400) and SAA 0–25 μg/mL (ref 0–20). Ocular effects of the subjconjucntival injection were similar between MSC and control eyes on SPOTS grading system, with conjunctival hypermia, chemosis and ocular discharge noted bilaterally, which improved without intervention within 14 days. All other ocular parameters were unaffected throughout the study. Necropsy and histopathology revealed no evidence of systemic inflammation. Ocular histopathology was similar between MSC and control eyes. Fluorescent imaging analysis did not locate MSCs. Immunohistochemistry did not identify intact MSCs in the conjunctiva, but GFP-labeled cellular components were present in conjunctival phagocytic cells.

**Discussion:**

Allogeneic pIC-activated conjunctival MSC injections were well tolerated. GFP-labeled tracking identified MSC components phagocytosed by immune cells subconjunctivally. This preliminary safety and tracking information is critical towards advancing immune conditioned cellular therapies to clinical trials in horses.

## Introduction

Equine recurrent uveitis (ERU) is a T-helper type I immune-mediated condition that affects up to 25% of horses in the United States (1) and is the most common cause of blindness, cataracts and glaucoma (2–5). ERU results when disruption of the blood-ocular barrier allows CD4+ T-lymphocytes to enter the eye, leading to recurring or persistent episodes of intra-ocular inflammation through recognition of self-antigens (6–8). The inciting causes for development of ERU are likely multifactorial and remain unclear in many cases, but may involve genetic predisposition in certain breeds (e.g., Appaloosas, Warmbloods, Icelandics) or infection with *Leptospira* spp. (5, 9–12). Current treatments for ERU include a combination of topical and systemic anti-inflammatories, atropine, antibiotics (if active *Leptospira* spp. infection is suspected) and long-term suprachoroidal cyclosporine implantation (2, 5). However, ERU remains challenging to manage, with a poor long-term prognosis for vision retention as 46.9% of eyes diagnosed eventually lose sight (2). Therefore, novel therapeutic strategies to combat the pro-inflammatory aspect of ERU are necessary.

Mesenchymal stem cells (MSCs), or mesenchymal stromal cells, are progenitor cells derived from a variety of tissues such as bone marrow, adipose tissue, umbilical cord blood, and peripheral blood (13). MSCs have the potential to modulate various cells of the immune system upon implantation, and have been evaluated as a potential treatment for many inflammatory diseases as well as to improve tissue healing (14–17). Allogeneic MSCs (from a donor cell line) can be administered to alleviate concerns including delay required for isolation and culture and individual patient characteristics (e.g., age, genetic factors, disease states) that may make autologous use less desirable (18, 19). While MSCs have shown promising immunomodulatory capacity *in vitro*, there are limited controlled *in vivo* studies critically evaluating their safety and treatment potential.

Although much of veterinary medicine has previously focused on the potential of MSCs to improve orthopedic injury outcomes, MSC therapy may be beneficial in ocular conditions such as equine recurrent uveitis. Loss of vision associated with ERU is a serious consequence for a horse and can result in enucleation, rehoming, or euthanasia (2). Recent evidence supports a potential role for subconjunctival MSC to treat other ocular conditions in horses, including immune mediated keratitis (IMMK) and corneal ulceration (20–24). Immune mediated keratitis is a local inflammatory disease that can reduce vision through scarring, secondary infections and corneal edema (20–22). A case series using MSCs injected subconjunctivally for IMMK reported clinical improvement and no side effects in four horses (23). Subconjunctival injections of MSC have been used to promote healing in corneal ulcers by private practitioners with anecdotal positive effects. MSCs in co-culture and MSC culture supernatant had positive effects on corneal epithelial cell healing *in vitro* (24).

Administering MSCs subconjunctivally has the potential to deliver secreted immunomodulatory factors locally and alter the immune response via direct cell interaction. Horses with ERU have an altered systemic T-cell profile with CD4+ T-cells expressing high levels of IFN-γ, and *in vitro* MSCs co-cultured with T-cells can reduce the T-cell expression of IFN-γ (6). One mechanism that MSCs exert their impacts on T-cells is through the secretions of prostaglandin E_2_ and other beneficial soluble immunomodulatory factors (13, 25, 26). Administering MSCs subconjunctivally has the potential to allow these soluble factors to reach the uvea in higher concentrations than systemic administration alone. MSCs also exert some of their immunomodulatory benefits via direct cell interaction, hence being placed in the local ocular environment allows for the greatest potential benefit (27).

Research assessing reciprocal interactions between the environment and MSCs has revealed that MSCs behavior and secretory profiles depend upon the local environmental signals that they receive (28–31). MSC activation through toll like receptors (TLRs), such as TLR3 agonist polyinosinic:polycytidylic acid (pIC), has been demonstrated to enhance MSCs immunomodulatory and antimicrobial potential without producing a more antigenic MSC for allogeneic use (28, 29, 32–34). Activating MSCs prior to therapeutic use has the potential to produce a more consistent and efficacious response to therapy. However, no controlled studies have been performed to evaluate adverse responses and determine how long MSCs stay in the injection site to exert a potentially beneficial effect on the ocular environment when injected via subconjunctival route. Additional safety and cell survival information is necessary prior to clinical trials to further investigating MSC therapy to treat ERU.

Therefore, to harness the full capacity of MSCs for ocular regenerative approaches, an improved understanding of the safety and duration of cell survival locally is indicated. In this study, we sought to evaluate the systemic and ocular effects of pIC-activated MSCs injected subconjunctivally in a series of three treatments and to evaluate the fate of these cells 14 days after the final treatment. Our hypothesis was that subconjunctival injections with pIC-activated MSCs would be well tolerated and have minimal adverse ocular or systemic effects in normal horses and would remain in ocular tissues for at least 14 days.

## Methods

### Study overview

**Figure 1:**
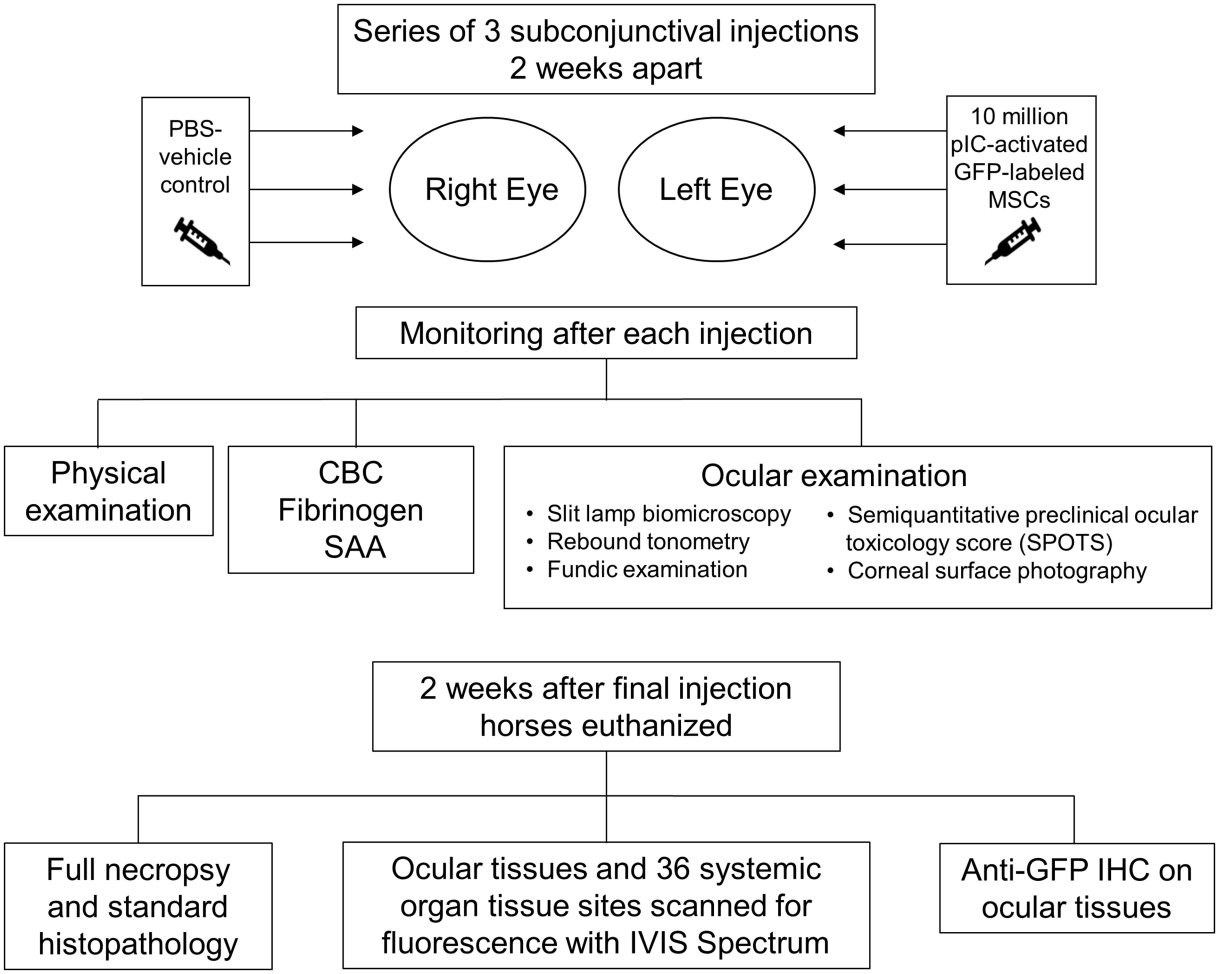
Study overview.

The bulbar conjunctiva of the left eye of two horses was injected with 10×10^6^ pIC-activated GFP-labeled MSCs from one donor three times at two-week intervals, while the conjunctiva of the right eye was injected with a vehicle (saline) control (Figure 1). Horses received physical and ophthalmic exams every 1 to 3 days. Systemic inflammation was assessed via complete blood count (CBC), fibrinogen, and serum amyloid A (SAA). Horses were euthanized 14 days following final injection. Full necropsy and histopathology were performed to examine ocular tissues and 36 systemic organs for MSC presence via IVIS Spectrum. Anti-GFP immunohistochemistry was performed on ocular tissues.

### Horses

The two adult horses (17-year-old female draft horse and 8-year-old male Quarter Horse) that were MSC recipients in this study were scheduled for euthanasia due to quality-of-life concerns related to progressive orthopedic and neurologic disease. Both draft horses and quarter horses are affected with ERU, thus both are targets of potential treatment populations (5). The two horses were housed at the Center for Equine Health (CEH). All procedures were approved by the Institutional Animal Care and Use Committee and the Clinical Trials Review Board at the University of California, Davis, protocol #21651.

Other than advanced orthopedic and neurologic disease, horses were determined to be systemically healthy based on physical examination, ocular examination, and bloodwork (CBC and biochemistry profile within normal limits). Detailed ocular examinations were performed by a board-certified veterinary ophthalmologist to document baseline findings. Horses did not receive any ocular medications, vaccinations or treatment with systemic anti-inflammatories (such as non-steroidal anti-inflammatories or steroids) within four weeks of study enrollment.

### MSC culture and GFP transduction

The MSCs for the study were previously isolated for other studies and derived from a single equine donor’s adipose tissue (healthy 1-day-old female Quarter Horse) that was born to a mare in the teaching herd and these samples were collected *in vivo* within 24 h of birth. The MSCs were cryopreserved as previously described and had been stored for 10 years in liquid nitrogen prior to this study (35). Equine MSCs were thawed and analyzed on flow cytometry for purity and identity using CD44 (clone CVS18; AbD Serotec, Raleigh, NC, United States), CD29 (clone 4B4LDC9LDH8; Beckman Coulter, Brea, CA, United States), CD90 (clone DH24A; VMRD, Pullman, WA, United States), as well as confirmed to be negative for F6B (white blood cell label; gift of Dr. Jeffrey Stott, UC Davis) (see supplemental figures from previous study) (36). Major histocompatibility haplotyping was not performed due to the terminal nature of this study.

The MSCs were transduced with eGFP/luciferase (pCCLc-MNDU3-LUC-PGK-EGFP-WPRE; gift of Dr. Jan Nolta, UC Davis) in a previous study (36). Briefly, equine MSCs were thawed and cultured for three days prior to transduction in complete stem cell medium (Dulbecco’s Modified Eagle’s Medium (DMEM), Gibco catalog #11885084 with 10% FBS and 1% penicillin–streptomycin, Gibco catalog # 15140122). Cells were trypsinized, pelleted, resuspended in transduction medium (DMEM, protamine sulfate 10 μg/mL), and plated in a T75 flask. GFP/luciferase lentivirus was added (multiplicity of infection (MOI) = ~10) and the cells were incubated overnight. After 24 h, two volumes of complete stem cell media were added to the flask. Cells were passaged and counted three days after transduction and again five days later. The eGFP efficiency was determined by fluorescent microscopy and flow cytometry at both time points (see figures from previous study) (36). Cells were then frozen and stored in liquid nitrogen as previously described (36).

The MSCs were thawed and cultured for 48 h prior to injection at each time point. They were trypsinized, enumerated and confirmed to be >90% viable prior to each injection based on trypan blue dye exclusion. They were confirmed to be negative for endotoxin (PYROGENT Plus LAL Gel Clot Assay; Lonza, Walkersville, MD, United States) and mycoplasma (Myco-Scope PCR Kit; Genlantis, San Diego, CA, United States) (Supplementary Figure S1). MSCs were activated by incubating with 10 ug/ml Polyinosinic:polycytidylic acid (pIC; Sigma-Aldrich, St. Louis, MO) for 2 h at 2×10^6^ cells/ml in complete stem cell medium, then washed three times with Dulbecco’s Phosphate Buffered Saline (DPBS) and resuspended in DPBS at a volume of 0.5 mL (10×10^6^ cells total) for injection. The two washes of DPBS would reduce the FBS on the cell surface but would not remove the intracytoplasmic FBS or FBS adhered to the cell surface (37). The number of MSCs used for injection (10×10^6^) was selected based on the range that is typically used for local delivery and the frequency on the lower end of typical multiple administrations to increase the chance of successful tracking (23, 38, 39).

### MSC injection

Horses were sedated using detomidine (0.01 mg/kg intravenously) and butorphanol (0.01 mg/kg intravenously). Auricular palpebral and supraorbital nerve blocks were performed by infiltrating the subcutaneous and peri-orbital tissue (2% lidocaine, approximately 2 mL per site). Topical anesthetic was applied to surface of the eye to provide additional anesthesia (Tetracaine 0.5%, approximately 0.5 mL). The conjunctiva was lavaged with dilute povidine-iodine (1:100 dilution).

Subconjunctival injection of 10 × 10^6^ pIC activated GFP-labeled MSCs diluted in DPBS to a total volume of one mL was drawn into a syringe with an 18 gauge needle and injected under the dorsal bulbar conjunctiva of the left eye with a 25 gauge needle (Supplementary Video S1). Subconjunctival injection of 1 mL of DPBS (vehicle control) was performed under the dorsal bulbar conjunctiva in the right eye by a boarded ophthalmologist (BCL or BCM). Each horse received a series of three treatments 14 days apart.

### Clinical monitoring

Complete physical examinations and ophthalmic examinations were performed prior to and after each MSC injection (day 0, day 1, day 3, day 7, and day 14). These days were selected to monitor for changes early on following injection similar to a joint flare (which typically occurs within 24–48 h following intra-articular MSC injection), as well as in consultation with the veterinary ophthalmologists’ in the study (40). Horses were observed daily for comfort but only examined on the days described. Ophthalmic examinations day 0, day 1, and day 14 were performed by a boarded veterinary ophthalmologist (BCL or BCM) and day 3 and day 7 examinations by an experienced equine clinician (JMC). Corneal surface photography was performed at each exam to ensure ocular surface findings were scored consistently. Peripheral blood (10 mL) was collected via the jugular vein then placed in an Ethylenediaminetetraacetic acid (EDTA) tube on day 0, day 1, day 3, and day 7, and day 14 prior to and after each MSC injection. Serum amyloid A (SAA), an acute phase protein and marker of acute inflammation was measured on day 0, day 1, day 3, day 7, and day 14 using a commercial quantitative lateral flow assay and reader (Stable – lab EQ-1 Zoetis, normal is less than 20 ug/mL) (41). Complete blood counts and fibrinogen, another marked of inflammation were performed by the William R. Pritchard Veterinary Medical Teaching Hospital (VMTH), UC Davis on day 0, day 3, day 7, and day 14.

### Ophthalmic examinations

Initial and subsequent ophthalmic examinations included slit lamp biomicroscopy evaluation, rebound tonometry intraocular pressure (IOP) measurement, fundic examination, fluorescein stain, and semiquantitative preclinical ocular toxicology scoring (SPOTS) (42), which includes conjunctival hyperemia score on all horses. A local nerve block was performed of the auriculopalpebral nerve and the supraorbital nerve (2% lidocaine, approximately 2 mL per site) to facilitate ocular examination. The same individual performed the rebound tonometry with a Tonovet Plus (iCare) measurements at all timepoints for consistency. Corneal surface photography was performed at each exam.

### Necropsy and systemic histopathology

Following humane euthanasia with 120 mLs of pentobarbital intravenously, a complete necropsy and tissue collection was performed immediately by a board-certified veterinary pathologist. Histopathology from systemic organs (Supplementary material 1) was evaluated to screen for any evidence of inflammation in response to MSC injection. Ocular histopathology was performed as described below.

### Ocular histopathology

Whole globes with conjunctiva and eyelids still attached were preserved in 10% neutral-buffered formalin. The samples were trimmed by a boarded pathologist to preserve orientation and paraffin wax embedded. All samples were sectioned at 4 μm and processed for routine histology then either stained with hematoxylin and eosin (H&E) according to a standard protocol for examination by light microscopy or prepared for immunohistochemistry (IHC) labeling. H&E-stained sections were evaluated for the severity, type, and location of conjunctival inflammation and the severity of inflammation was scored in accordance with a previously reported feline conjunctivitis scoring system adapted to equine tissues (Table 1) (43, 44).

**Table 1:** Conjunctival inflammation scoring scheme for ocular histopathology.

Score	Description
Grade 0 (normal)	Inflammatory cells are absent or there are sparsely scattered, individual inflammatory cells, and 1 to 2 subepithelial lymphoid follicles
Grade 1	Scattered aggregates or diffusely distributed low numbers of inflammatory cells
Grade 2	Focally large or diffusely moderate numbers of inflammatory cells with or without mild distortion of tissue architecture
Grade 3	Diffuse infiltration or effacement of the mucosa by large numbers of inflammatory cells with distortion of tissue architecture

### *Ex vivo* fluorescent imaging

Tissue collection was performed immediately following euthanasia to minimize autofluorescence. The globe and surrounding conjunctiva, as well as sections from all major organs and lymph nodes were imaged with the IVIS Spectrum (Supplementary material 2) (45). The IVIS Spectrum (Perkin Elmer) is a high sensitivity optical imaging system which at low magnification allows for a wide field setting to screen organ sections. All images were performed under the same standard settings to capture the GFP peak, 465/520 (excitation/emission), followed by 500/540 (excitation/emission), with medium binning at 8×8 pixels, utilizing a 16-bit (65,536 gray level) detector. Every tissue was imaged using consistent settings. Analysis was performed in Fiji/ImageJ; manual tracing of the conjunctiva and sclera was performed by a masked observer and average GFP signal was determined for each tissue and reported as Relative Fluorescence Units (RFU) (46).

### Immunohistochemistry

The IHC protocol was optimized with dissected whole globes with conjunctiva and eyelids still attached that were injected with 7.5 ×10^5 GFP transfected MSCs in 0.5 mL PBS postmortem and then this was used as a positive control for IHC. Briefly, formalin fixed paraffin embedded sections were deparaffinized in xylene and exposed to decreasing concentrations of ethanol followed by quenching solution (50 mL of 1X PBS, 500 μL of 10% Sodium Azide, 500 μL of 30% Hydrogen Peroxide). Antigen retrieval was performed by incubating the slides in citrate buffer in a pressure cooker at approximately 120 ^o^ F for 5 min and then allowed to depressurize over 30 min. Sections were blocked with 10% normal horse serum. Sections were incubated with monoclonal mouse anti-GFP primary antibody (Sigma-Aldrich, clone GT859) at a concentration of 1:100 in 10% normal horse serum for 1 h at room temperature followed by three washes. Secondary anti-mouse/rabbit antibody VECTASTAIN ELITE ABC anti-mouse/rabbit, R.T.U. (Ready to Use) HRP Immunodetection Kit (Novus Bio, PK-7200-NB) was applied and incubated for 30 min at room temperature followed by 3 washes. Vector NovaRed Substrate Kit (Vector Laboratories, SK-4800) was prepared according to manufacturer directions and applied individually to each slide and rinsed after color developed in the positive control. The IHC slides were counterstained with Modified Mayer’s Hematoxylin (Richard Allan Scientific), then washed in tap water. Slides were dehydrated and cover slipped using Shandon-Mount mounting medium (Thermo Fisher Scientific).

Histological images were viewed and acquired on Olympus BX40 equipped with Olympus DP72 camera and cellSens XV image processing software. All IHC images were captured at 4x and 40x magnifications.

## Results

### Clinical monitoring

**Figure 2:**
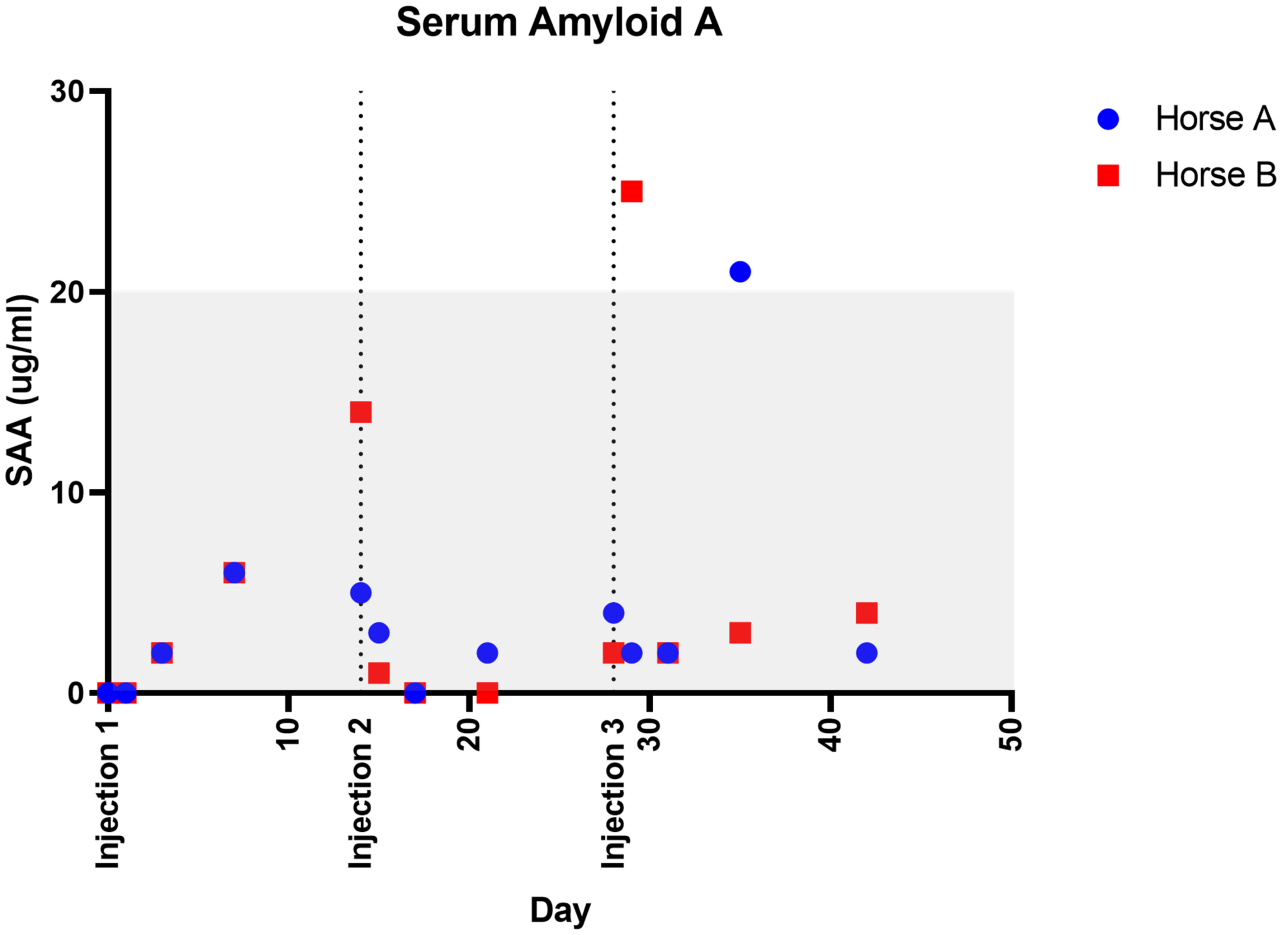
Serum amyloid A values over time. Injections were performed at 0, 14, and 28 days. Shaded area represents normal reported reference values, <20 ug/ml.

There was no change in general physical examination parameters (temperature, heart rate, respiratory rate, digital pulses, gastrointestinal motility borborygmi) following injection or at any time point over the course of the study (Supplementary Table S1). Complete blood count values were either within the laboratory reference ranges or slightly outside range (most notably Horse A slightly lymphopenic) throughout the study (Supplementary Table S2). Fibrinogen values ranged between 100–300 mg/dL (laboratory ref. range 100–400) (Supplementary Table S2) and SAA values ranged between 0–25 ug/ml (ref range 0–20) (Figure 2) (41).

### Ophthalmic examinations

**Figure 3:**
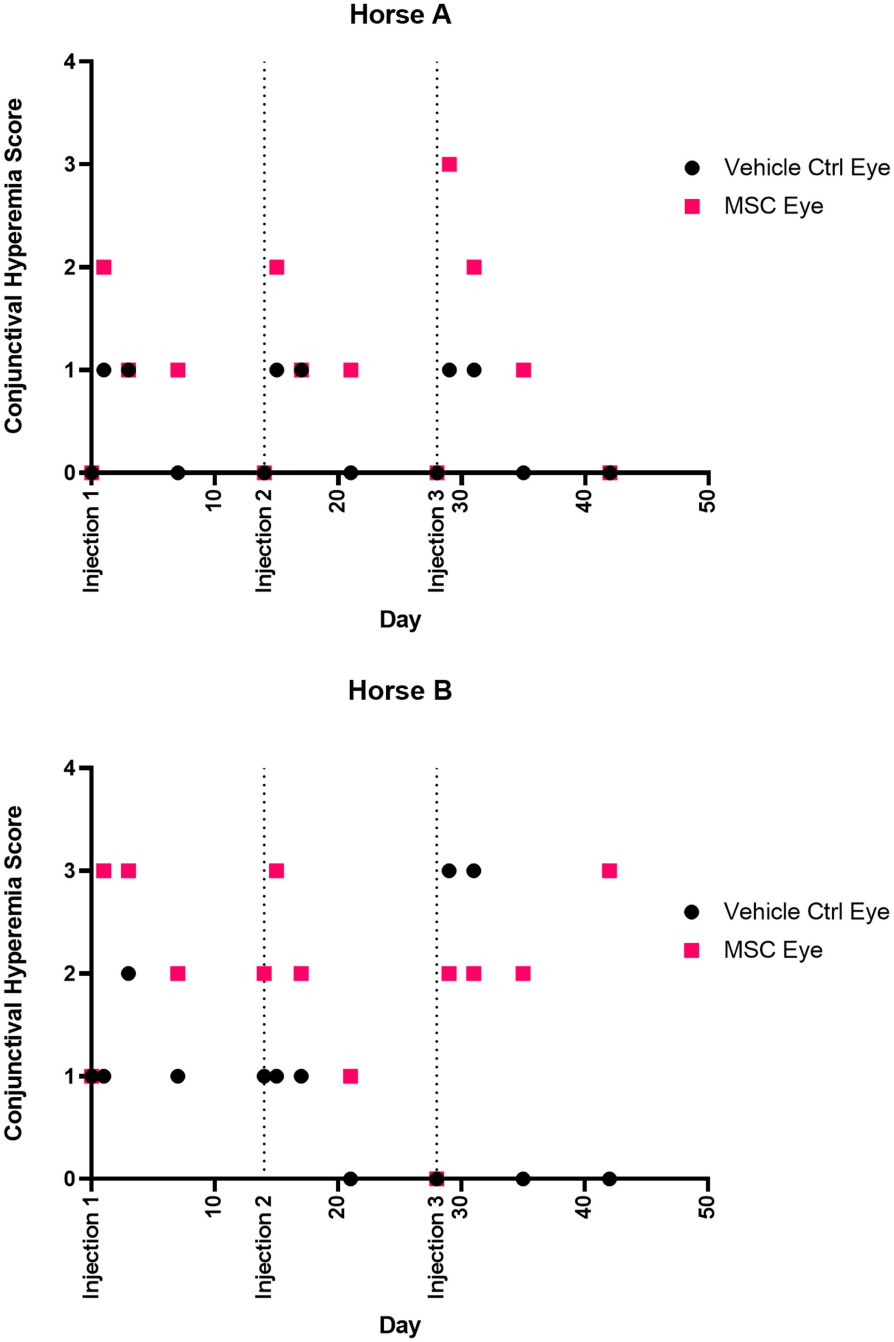
Conjunctival hyperemia score over time. Injections were performed at 0, 14, and 28 days. Eyes were scored according to the semiquantitative preclinical ocular toxicology score (SPOTS) system. Scores on days 0, 14, and 28 were prior to injection.

**Figure 4:**
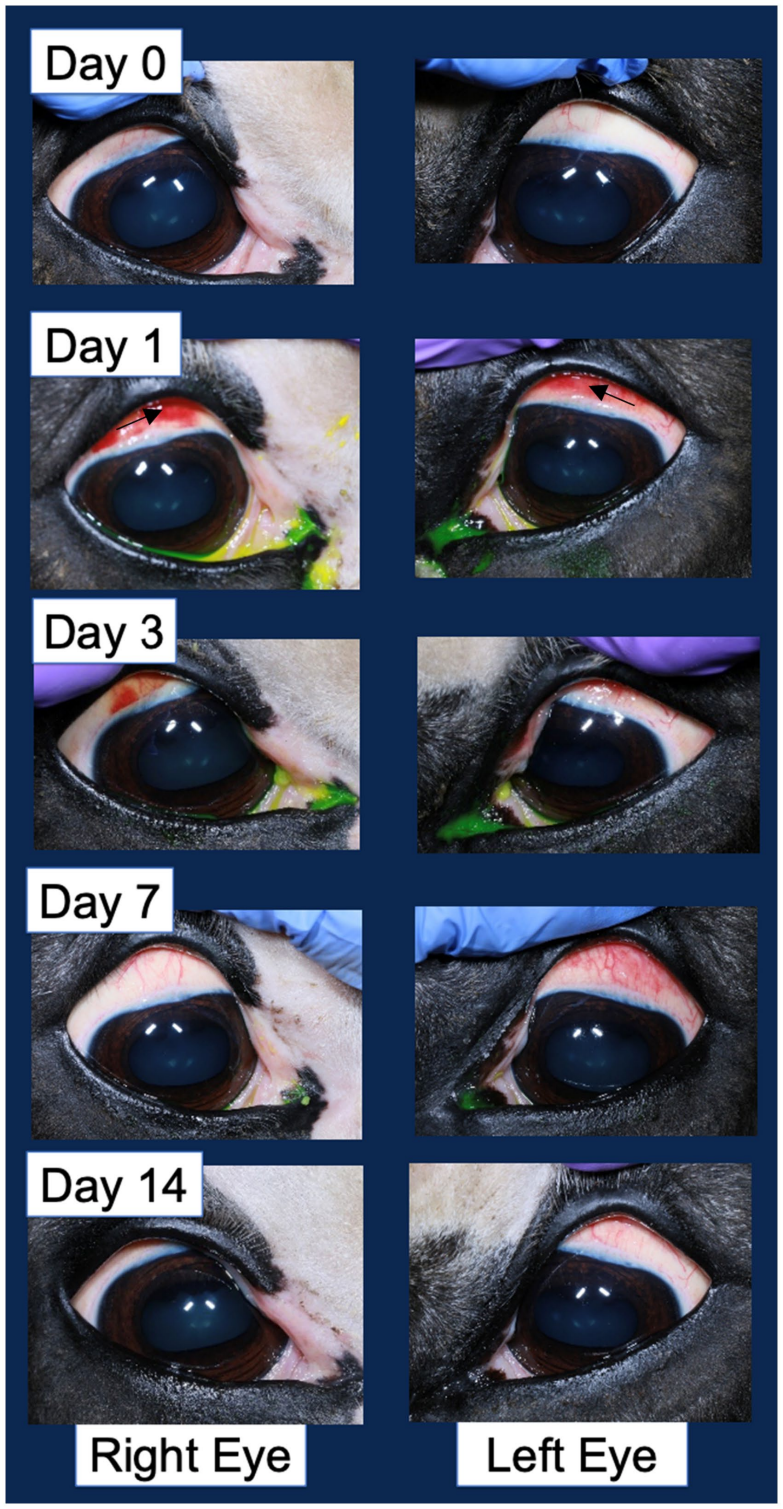
Example corneal surface photography documenting the ocular changes following injection in both the vehicle control injected right eye and pIC-activated GFP-labeled MSC injected left eye. The most significant change of note is the hyperemia (redness associated with blood vessel engorgement) of the bulbar conjunctiva. An arrow in the second row of images denotes the area of hyperemia.

Ocular effects of the subconjunctival injections were similar between the MSC and PBS eyes on the SPOTS grading system, and included conjunctival hyperemia, chemosis and ocular discharge (Figure 3; Supplementary Table S3). These ocular effects were documented with ophthalmic photography (Figure 4). Intraocular pressure assessed by rebound tonometry for Horse A ranged between 15–20 mmHg for the right control eye and between 15–22 mmHg for the left MSC injected eye. For Horse B intraocular pressure ranged between 12-22 mmHg for the right control eye and 14-19 mmHg for the left MSC injected eye. Normal horse Tonovet Plus measurements can range from 13.2–33.2 mmHg, when gold standard manometer pressures range between 20–30 mmHg (47). All other ocular parameters remained stable throughout the study. Ophthalmic clinical signs improved without intervention by day 14 following injection.

### Necropsy and histopathology

Standard necropsy and histopathology of standard systemic tissue set revealed no evidence of systemic inflammation or toxicity.

### *Ex vivo* fluorescent imaging

**Figure 5:**
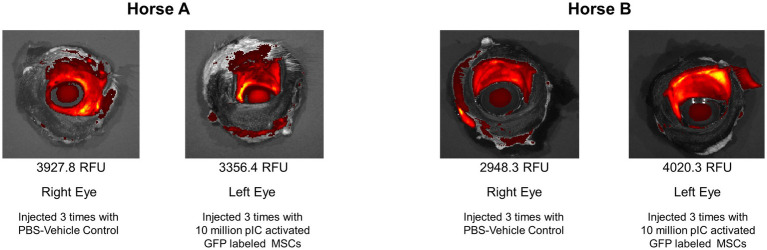
IVIS Spectrum images of eyes. The mean fluorescent intensity was calculated over the conjunctiva and sclera. Horse A was 3927.8 relative fluorescent units (RFU) for the right (control) eye and 3356.4 RFU for the left (MSC) eye, whereas Horse B was 2948.3 RFU for the right (control) eye and 4020.3 RFU for the left (MSC) eye.

Significant autofluorescence was visualized in all samples, with higher reads of fluorescence on tissues with a moist mucosal surface. No regions of local increased fluorescence within the tissues were noted that could be attributed to GFP labeled MSCs. When comparing the left and right whole globes, the mean intensity was calculated over the conjunctiva and sclera. Maximal detected values were approximately 10,000 RFU and background levels were approximately 800 RFU. Horse A was 3927.8 RFU for the right (control) eye and 3356.4 RFU for the left (MSC) eye, whereas Horse B was 2948.3 RFU for the right (control) eye and 4020.3 RFU for the left (MSC) eye (Figure 5).

### Ocular histopathology

**Figure 6:**
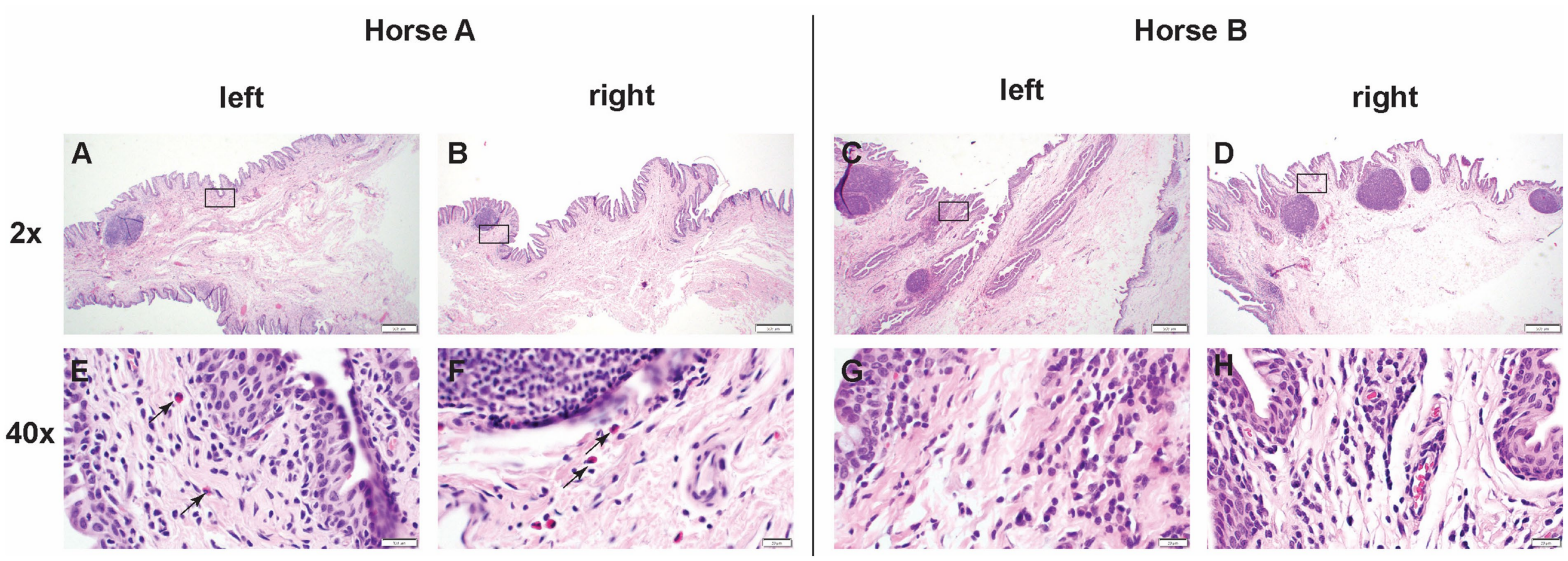
Low (2x) and high (40x) magnification images of hematoxylin and eosin sections from positive control injected conjunctiva and study pIC-activated GFP labeled MSC injected conjunctiva. The rectangle on low magnification image indicates the area captured on high magnification. Horse A’s right (control) conjunctiva and left (MSC injected) conjunctiva, as well as horse B’s right (control) and left (MSC) conjunctiva were all scored as grade 1 (scattered aggregates or diffusely distributed low numbers of inflammatory cells). The inflammation was predominantly lymphoplasmacytic and bilaterally symmetrical in both eyes. In horse A, rare eosinophils were present among lymphocytes and plasma cells. Scale bar = 400 μm on low (2x) magnification captures and 20 μm on high (40x) magnification captures.

Left and right eyes of both Horse A and B ocular histopathology inflammation were scored as 1 (scattered aggregates or diffusely distributed low numbers of inflammatory cells) (Table 1). The inflammation was predominantly lymphoplasmacytic and bilaterally symmetrical in both eyes. In horse A, rare eosinophils were present among lymphocytes and plasma cells (Figure 6).

### Immunohistochemistry for GFP labeled MSCs

**Figure 7:**
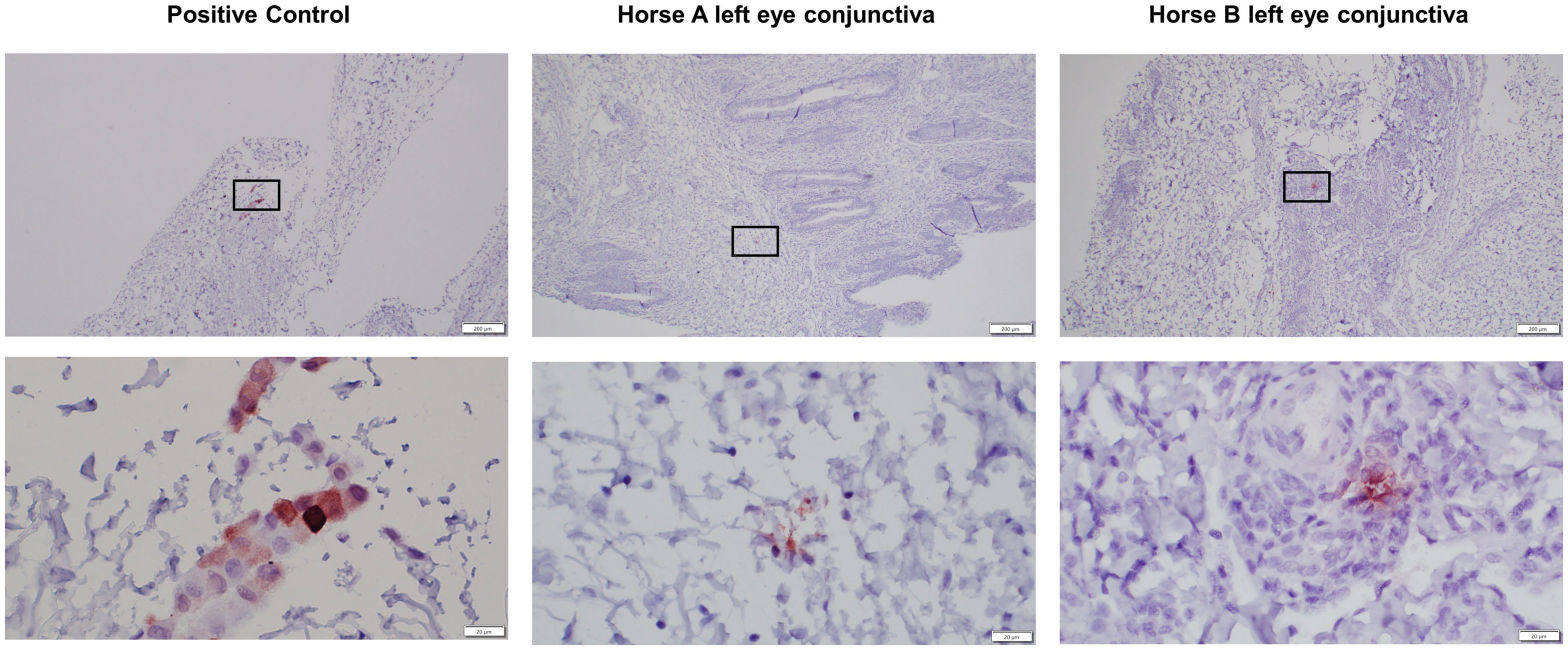
Low (4x) and high (40x) magnification images of GFP immunohistochemical sections of conjunctiva from positive control injected eyes and study pIC-activated GFP labeled MSC injected eyes. The rectangle on low magnification image indicates the area captured on high magnification. Positive control is post-mortem GFP-labeled MSC-injected conjunctiva slides, individual cells consistent with the post-mortem injection sites are clearly labeled. The left eyes, injected three times with the pIC-activated GFP-labeled MSCs, have rare, pinpoint, intracellular immunolabeling present suggestive of MSCs phagocytosed by tissue macrophages or dendritic cells. There was no immunolabeling on the right control eyes (not pictured). Scale bar = 200 μm on low (4x) magnification captures and 20 μm on high (40x) magnification captures.

In the positive control post-mortem GFP-labeled MSC-injected conjunctiva slides, individual cells consistent with the post-mortem injection sites are clearly labeled (Figure 7). In the PBS vehicle control injected eyes, there was no staining detected. In the pIC-activated GFP-labeled MSCs injected eyes, there was no bright individual cell staining consistent with intact MSCs; however, there was some faint staining present suggestive of MSCs phagocytosed by tissue macrophages or dendritic cells (Figure 7).

## Discussion

In this study we demonstrated that allogeneic pIC-activated GFP-plasmid-labeled MSCs from a single donor horse injected three times at two-week intervals into the bulbar conjunctiva was well tolerated based on clinical parameters and histopathology in two horses. GFP-labeled MSC tracking with immunohistochemistry suggested that while intact MSCs were not present after 14 days, GFP was detected within phagocytic cells, suggesting phagocytosis of GFP labeled MSCs that could result in immunomodulation (48). This preliminary safety and tracking information is critical to move immune conditioned cellular therapies injected via subconjunctival route into equine clinical trials to evaluate potential disease modifying effects in equine recurrent uveitis.

In the past, MSC treatments have previously gone to clinical application with minimal safety investigation nor proof of efficacy, due to an initial lack of regulation in veterinary species (49). This study provides controlled data to document a lack of adverse events at the clinical and histological level that would not be available from patients enrolled in a clinical trial. While there have been some promising results of MSCs in equine orthopedic applications (38, 40, 50), other reports have failed to demonstrate a benefit from MSC treatment or efficacy is inconsistent (37, 51). As of 2015, MSCs in veterinary use have been classified as a drug by the FDA and require an Investigational New Animal Drug (INAD) submission and report for clinical use (49). Thus, as the standard for proof of efficacy has been elevated, each new indication for veterinary MSC use will necessitate a safety and efficacy trial. While the sample size of this study is small and further monitoring in a larger patient population and over greater study duration is indicated, these findings provide initial data towards further evaluation of MSCs for clinical ocular indications.

An immune response to allogeneic MSCs has been previously demonstrated (37, 52, 53), although efficacy following repeated injections remains to be fully assessed. This study implemented allogeneic MSC due to benefits including availability for immediate administration following screening for infectious diseases and known potency, while autologous cell preparations carry concerns of failure of MSC isolation and poor health of the MSCs due to patient age and genetic background (18, 54). Minimal systemic inflammatory response was documented in this study in response to MSC injection, as all fibrinogen values were within the laboratory’s normal reference range and only 1 timepoint for each horse was slightly above reference range for SAA (Figure 2; Supplementary Table S2). Both fibrinogen and SAA are acute phase proteins produced by the liver in response to systemic inflammation. SAA has a more rapid response to inflammation with marked increases within 6 h of inflammatory stimulus, whereas fibrinogen takes 24 h to respond to an inflammatory stimulus (41). The mild increase in SAA in Horse B twenty-four hours about the 3rd injection may be due to some transient inflammation following injection, however it cannot be ruled out that this was due to another inflammatory stimulus in the environment or even simply a variation of normal. Major histocompatibility typing was not utilized in this study due to the terminal nature, but can be used to assess whether donor MSCs are a complete mismatch or partial match, with the potential to reduce the adaptive immune response (52, 53).

This study evaluated MSCs activated with pIC, a TLR-3 agonist, which has been shown to increase MSC immunomodulatory capabilities without producing a more antigenic MSC for allogeneic use (28, 29, 32–34, 55–58). Activation with pIC *in vitro* can result in a more homogenous MSC immunomodulatory response, suggesting pIC activated MSCs could perform more consistently *in vivo* (28, 29, 58). Given the greater potential for immune response to allogeneic MSCs, it may be even more essential that they are as potent as possible through pIC activation, so they can actualize their benefits prior to being targeted by the host immune system (29). Activated MSCs were utilized in this study to insure that while they have superior immunomodulatory capabilities *in vitro* (28, 29, 59) and in other *in vivo* (56, 60) administrations, that they did not induce any intolerable adverse ocular responses.

In many MSC treatment protocols, multiple administrations of MSCs are recommended, however there is often minimal data to support the timing interval (23, 38, 56, 60). In several studies where MSC tracking has been reported, cell presence is markedly reduced within 14 to 21 days (61, 62) which has led to the paradigm shift in thinking that the proposed benefit of MSC is through recruitment and modulation of immune effector cells rather than differentiation into local cell types (55, 63). MSCs labeled with GFP can maintain this fluorescent marker for prolonged periods of time *in vitro* (14 weeks documented) and in vivo (up to 90 days documented) (61, 64). In this study, we attempted to track the GFP labeled MSCs using two modalities, *ex vivo* whole tissue fluorescent imaging with the IVIS Spectrum and immunohistochemistry of our target tissue the conjunctiva. Unfortunately, the background fluorescence in the GFP channel was too great to achieve meaningful tracking data with the IVIS Spectrum (65). The sclera is a collagen rich tissue, and distinguishing GFP signal from collagen autofluoresence is a known challenge (66), including specifically in IVIS images (67). From our preliminary optimization, the IVIS spectrum was able to detect as few as 50,000 GFP labeled MSCs injected post-mortem, but as the animals were euthanized 14 days following the third MSC injection, the cells did not appear clustered in concentrations sufficient for detection. When evaluating the conjunctiva for the presence of GFP labeled MSCs with our anti-GFP antibody, it appeared that the MSCs had been phagocytosed by immune cells present in the conjunctiva. While we cannot say this with certainty, there was no non-specific staining in the vehicle control injected eye, and the low intensity staining in the immune cells was only seen in the eye injected with MSCs. It has been proposed that one way MSCs induce immunomodulation is through phagocytosis by macrophages and dendritic cells (55). If the main way MSCs induce immunomodulation in the conjunctiva is through being phagocytosed by local innate immune cells, the fact that their cellular components were still present in these cells at 14 days would suggest this would be an appropriate treatment interval to evaluate in a clinical trial.

A strength of this study was the vehicle control injections of the contralateral eye, allowing for a more accurate assessment of the local response to the needle insertion and volume injection given individual variability and the small sample size. While the MSC injected eye did have higher scores for conjunctival hyperemia following injection in both Horse A and B, this resolved by 14 days in injections 1 and 2. Following injection 3 in Horse B, the conjunctival hyperemia did not resolve within the 14 days prior to study conclusion in the MSC injected eye, but there was not an appreciable difference in the conjunctiva inflammation histology between the MSC injected and on control injected eye. The local changes in hyperemia and chemosis seen in this study are similar to the expected inflammation induced by subconjunctival injection alone, suggesting local inflammation in response to both MSCs and vehicle control PBS injections (68). A larger study with more animals to allow for statistical analysis would allow for more definitive assessment of the safety of this treatment.

As a better understanding of MSCs has highlighted their ability to modulate the environment to reduce inflammation and improve healing, application of MSCs to treat other immune-mediated conditions such as equine recurrent uveitis represents a logical future direction of this work (14). Additionally, administration of MSCs into the conjunctiva is relatively non-invasive and requires only standing sedation, compared to the general anesthesia required to place a cyclosporine implant in the suprachoroidal space to aggressively treat ERU (2). Next steps in this work will be to evaluate activated MSCs ability to improve clinical signs in patients diagnosed with ERU that are non-responsive to current management techniques.

## Conclusion

This preliminary safety study demonstrated allogeneic pIC-activated MSC injection into the equine conjunctiva was well-tolerated in two horses, providing essential initial safety information prior to clinical application in horses. GFP-labeled MSC tracking with immunohistochemistry found GFP in phagocytes in the conjunctiva at 14 days, suggesting this would be a reasonable interval to evaluate multiple injections in a clinical trial. Activated MSCs have the potential to benefit patients with inflammatory ocular conditions, such as recurrent uveitis where novel treatments are needed. Future studies can move to evaluating comprehensive safety and the efficacy of activated MSCs to treat ocular conditions, with the knowledge that on initial investigation this therapy did not induce adverse events beyond injection alone.

## Data availability statement

The original contributions presented in the study are included in the article/Supplementary material, further inquiries can be directed to the corresponding author.

## Ethics statement

The animal study was approved by Institutional Animal Care and Use Committee and the Clinical Trials Review Board at the University of California, Davis, protocol #21651. The study was conducted in accordance with the local legislation and institutional requirements.

## Author contributions

JC: Conceptualization, Data curation, Formal analysis, Investigation, Project administration, Supervision, Writing – original draft, Writing – review & editing. BL: Methodology, Writing – review & editing. BM: Methodology, Writing – review & editing. NV: Formal analysis, Methodology, Writing – review & editing. JM: Formal analysis, Writing – review & editing. SD: Conceptualization, Writing – review & editing. KW: Conceptualization, Writing – review & editing. LP: Conceptualization, Methodology, Writing – review & editing.

## Supplementary material

SUPPLEMENTARY FIGURE 1 Endotoxin and mycoplasma detailed methods and results. To determine the MSC sample was free of endotoxin prior to injection, a 10 ul sample of cell suspension is diluted in 990 ul of reagent water, then 25 ul of this dilution is pipetted into each of the Charles River Endotoxin cartridges 4 reservoirs. The cartridge is then read on the Endosafe® nexgen-PTS™, a handheld spectrophotometer. For a valid test the coefficient of variation must be <25% between the sample replicates and recovery of the positive product control must be within 50-200% for a valid test result. All samples values measured <5 Endotoxin Units (EU)/mL. To determine the MSC sample was free of mycoplasma prior to injection, a 100 ul sample of supernatant from a tissue culture flask is incubated for 5 minutes at 950C and then added to the PCR reaction. The PCR reaction and cycling conditions are all performed as directed by the manufacturer’s guidelines in Abm’s Mycoplasma PCR detection kit, including a manufacturer provided positive control and negative control. The PCR products are run on a 1.2% agarose gel with a 6X fluorescent dye and imaged with a FluorChem E machine. An example mycoplasma PCR readout is shown; Lane 1: ladder, Lane 2: sample, Lane 3: Negative control, Lane 4: Positive Control.

SUPPLEMENTARY VIDEO 1 Injection of MSCs into bulbar conjunctiva.
