# Supplementary figures and images for: Preliminary evaluation of safety and migration of immune activated mesenchymal stromal cells administered by subconjunctival injection for equine recurrent uveitis
Source: Front Vet Sci. 2023 Dec 14;10:1293199. doi: 10.3389/fvets.2023.1293199 (PMC10757620; doi:10.3389/fvets.2023.1293199)

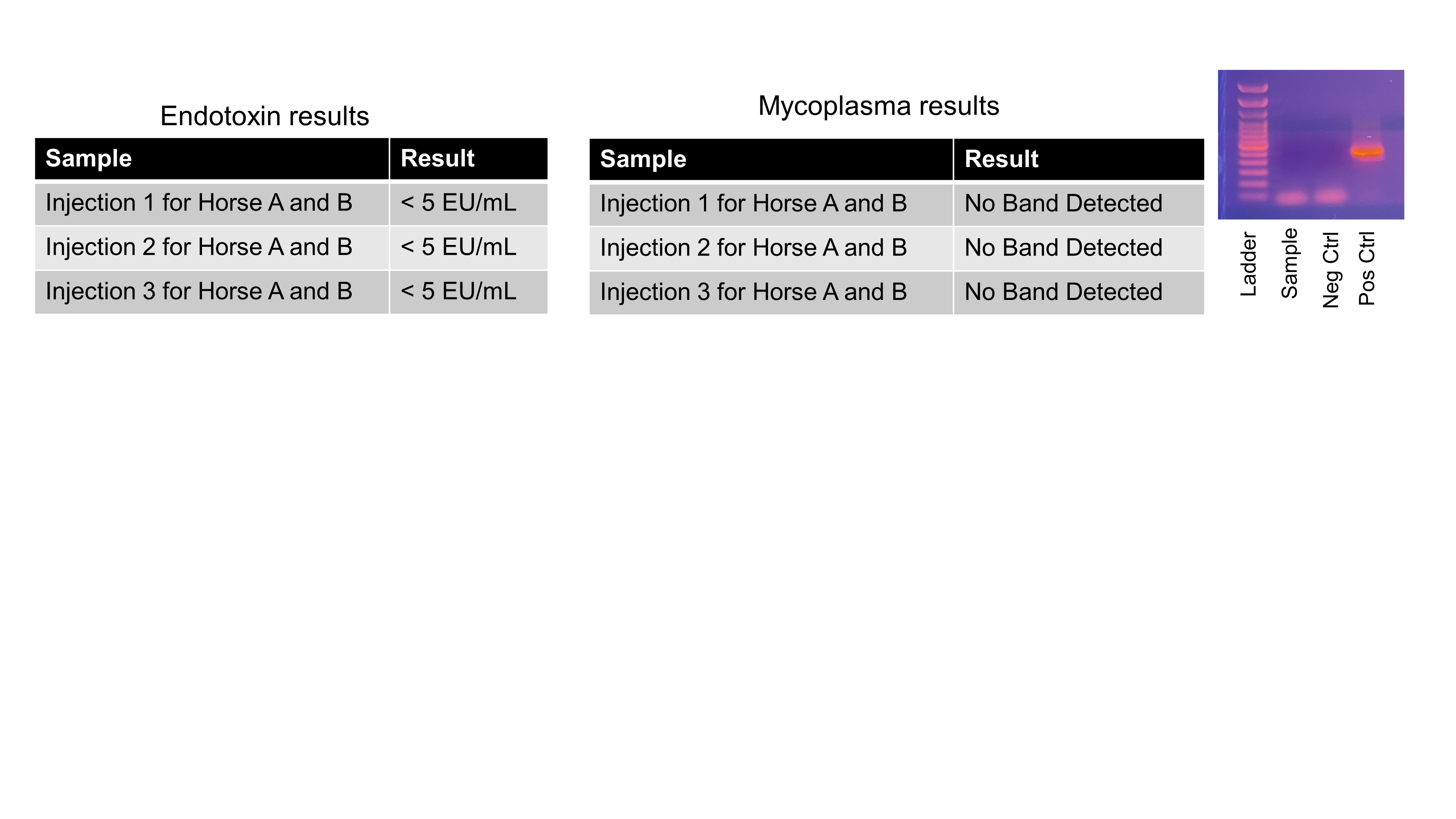

Supplement: SUPPLEMENTARY FIGURE 1 — Endotoxin and mycoplasma detailed methods and results. To determine the MSC sample was free of endotoxin prior to injection, a 10 ul sample of cell suspension is diluted in 990 ul of reagent water, then 25 ul of this dilution is pipetted into each of the Charles River Endotoxin cartridges 4 reservoirs. The cartridge is then read on the Endosafe® nexgen-PTS™, a handheld spectrophotometer. For a valid test the coefficient of variation must be <25% between the sample replicates and recovery of the positive product control must be within 50-200% for a valid test result. All samples values measured <5 Endotoxin Units (EU)/mL. To determine the MSC sample was free of mycoplasma prior to injection, a 100 ul sample of supernatant from a tissue culture flask is incubated for 5 minutes at 950C and then added to the PCR reaction. The PCR reaction and cycling conditions are all performed as directed by the manufacturer’s guidelines in Abm’s Mycoplasma PCR detection kit, including a manufacturer provided positive control and negative control. The PCR products are run on a 1.2% agarose gel with a 6X fluorescent dye and imaged with a FluorChem E machine. An example mycoplasma PCR readout is shown; Lane 1: ladder, Lane 2: sample, Lane 3: Negative control, Lane 4: Positive Control. [file Image_1.TIF]
