# Supplementary material for: Preliminary evaluation of safety and migration of immune activated mesenchymal stromal cells administered by subconjunctival injection for equine recurrent uveitis
Source: Front Vet Sci. 2023 Dec 14;10:1293199. doi: 10.3389/fvets.2023.1293199 (PMC10757620; doi:10.3389/fvets.2023.1293199)
Supplement: Supplementary file 3 [file Table_1.docx]

Supplemental Table 1: Summary of Physical Exam Parameters over time in horses following pIC-activated GFP-labeled

|  |  | **Injection 1** | |  |  | **Injection 2** | |  |  | **Injection 3** | |  |  |  |
| --- | --- | --- | --- | --- | --- | --- | --- | --- | --- | --- | --- | --- | --- | --- |
| Physical Exam | Horse | Day 0 | Day 1 | Day 3 | Day 7 | Day 14 | Day 15 | Day 17 | Day 21 | Day 28 | Day 29 | Day 31 | Day 35 | Day 42 |
| Attitude | A | BAR | BAR | BAR | BAR | BAR | BAR | BAR | BAR | BAR | BAR | BAR | BAR | BAR |
|  | B | BAR | BAR | BAR | BAR | BAR | BAR | BAR | BAR | BAR | BAR | BAR | BAR | BAR |
| Appetite | A | Good | Good | Good | Good | Good | Good | Good | Good | Good | Good | Good | Good | Good |
|  | B | Good | Good | Good | Good | Good | Good | Good | Good | Good | Good | Good | Good | Good |
| Manure | A | Formed | Formed | Formed | Formed | Formed | Formed | Formed | Formed | Formed | Formed | Formed | Formed | Formed |
|  | B | Formed | Formed | Formed | Formed | Formed | Formed | Formed | Formed | Formed | Formed | Formed | Formed | Formed |
| Temperature | A | 99.8 | 99.7 | 99.5 | 99.5 | 99.6 | 100.3 | 99.3 | 100.4 | 98.8 | 98.8 | 99.8 | 99.1 | 100.3 |
|  | B | 98.6 | 98.6 | 98.6 | 99.3 | 98 | 98.8 | 97.9 | 98.9 | 98.2 | 98.4 | 98.8 | 98.5 | 98.4 |
| Pulse | A | 36 | 44 | 36 | 36 | 40 | 42 | 30 | 40 | 40 | 40 | 36 | 40 | 36 |
|  | B | 44 | 40 | 40 | 40 | 40 | 50 | 50 | 40 | 40 | 48 | 48 | 36 | 42 |
| Respiratory rate | A | 24 | 28 | 20 | 40 | 24 | 30 | 20 | 18 | 20 | 30 | 20 | 16 | 18 |
|  | B | 20 | 20 | 20 | 20 | 20 | 20 | 24 | 14 | 20 | 20 | 16 | 20 | 18 |
| MM/ CRT | A | PM/ <2 | PM/ <2 | PM/ <2 | PM/ <2 | PM/ <2 | PM/ <2 | PM/ <2 | PM/ <2 | PM/ <2 | PM/ <2 | PM/ <2 | PM/ <2 | PM/ <2 |
|  | B | PM/ <2 | PM/ <2 | PM/ <2 | PM/ <2 | PM/ <2 | PM/ <2 | PM/ <2 | PM/ <2 | PM/ <2 | PM/ <2 | PM/ <2 | PM/ <2 | PM/ <2 |
| Gastrointestinal sounds | A | good x4 | good x4 | good x4 | good x4 | good x4 | good x4 | good x4 | good x4 | good x4 | good x4 | good x4 | good x4 | good x4 |
|  | B | good x4 | good x4 | good x4 | good x4 | good x4 | good x4 | good x4 | good x4 | good x4 | good x4 | good x4 | good x4 | good x4 |
| Digital pulses | A | WNL x4 | WNL x4 | WNL x4 | WNL x4 | WNL x4 | WNL x4 | WNL x4 | WNL x4 | WNL x4 | WNL x4 | WNL x4 | WNL x4 | WNL x4 |
|  | B | WNL x4 | WNL x4 | WNL x4 | WNL x4 | WNL x4 | WNL x4 | WNL x4 | WNL x4 | WNL x4 | WNL x4 | WNL x4 | WNL x4 | WNL x3, RF* |

BAR = Bright, alert, responsive

PM = pink moist mucus membranes

<2 = <2 seconds for capillary refill time

Good x4 = good gastrointestinal sounds all four quadrants

WNL x4 =digital pulses within normal limits on all four limbs

RF* = mild increase in digital pulses, no further diagnostics were performed as it was the day of humane euthanasia, but no lameness was observed

Supplemental Table 3: Summary of SPOTS over time in horses following pIC-activated GFP-labeled MSC injection

|  |  |  | **Injection 1** | |  |  | **Injection 2** | |  |  | **Injection 3** | |  |  |  |
| --- | --- | --- | --- | --- | --- | --- | --- | --- | --- | --- | --- | --- | --- | --- | --- |
| SPOTS Exam | Horse | Eye | Day 0 | Day 1 | Day 3 | Day 7 | Day 14 | Day 15 | Day 17 | Day 21 | Day 28 | Day 29 | Day 31 | Day 35 | Day 42 |
| PLR (0-3) | A | OD | 0 | 0 | 0 | 0 | 0 | 0 | 0 | 0 | 0 | 0 | 0 | 0 | 0 |
|  |  | OS | 0 | 0 | 0 | 0 | 0 | 0 | 0 | 0 | 0 | 0 | 0 | 0 | 0 |
|  | B | OD | 0 | 0 | 0 | 0 | 0 | 0 | 0 | 0 | 0 | 0 | 0 | 0 | 0 |
|  |  | OS | 0 | 0 | 0 | 0 | 0 | 0 | 0 | 0 | 0 | 0 | 0 | 0 | 0 |
| Conjunctival Hyperemia (0-3) | A | OD | 0 | 1 | 1 | 0 | 0 | 1 | 1 | 0 | 0 | 1 | 1 | 0 | 0 |
|  |  | OS | 0 | 2 | 1 | 1 | 0 | 2 | 1 | 1 | 0 | 3 | 2 | 1 | 0 |
|  | B | OD | 1 | 1 | 2 | 1 | 1 | 1 | 1 | 0 | 0 | 3 | 3 | 0 | 0 |
|  |  | OS | 1 | 3 | 3 | 2 | 2 | 3 | 2 | 1 | 0 | 2 | 2 | 2 | 3 |
| Conjunctival Chemosis (0-4) | A | OD | 0 | 1 | 1 | 1 | 0 | 0 | 0 | 0 | 0 | 0 | 0 | 0 | 0 |
|  |  | OS | 0 | 2 | 1 | 1 | 0 | 1 | 0 | 0 | 0 | 2 | 2 | 1 | 0 |
|  | B | OD | 0 | 1 | 1 | 1 | 0 | 0 | 0 | 0 | 0 | 1 | 1 | 0 | 0 |
|  |  | OS | 0 | 2 | 1 | 1 | 0 | 1 | 1 | 1 | 0 | 1 | 1 | 1 | 2 |
| Conjunctival Discharge (0-3) | A | OD | 0 | 1 | 2 | 2 | 0 | 0 | 1 | 0 | 0 | 0 | 1 | 1 | 0 |
|  |  | OS | 0 | 1 | 2 | 2 | 0 | 0 | 1 | 1 | 0 | 0 | 2 | 2 | 1 |
|  | B | OD | 0 | 2 | 2 | 1 | 0 | 0 | 0 | 0 | 0 | 0 | 0 | 0 | 0 |
|  |  | OS | 0 | 3 | 2 | 1 | 0 | 0 | 0 | 1 | 0 | 0 | 0 | 0 | 1 |
| Corneal Opacity (severity) (0-4) | A | OD | 1 | 1 | 1 | 1 | 1 | 1 | 1 | 1 | 1 | 1 | 1 | 1 | 1 |
|  |  | OS | 0 | 0 | 0 | 0 | 0 | 0 | 0 | 0 | 0 | 0 | 0 | 0 | 0 |
|  | B | OD | 0 | 0 | 0 | 0 | 0 | 0 | 0 | 0 | 0 | 0 | 0 | 0 | 0 |
|  |  | OS | 0 | 0 | 0 | 0 | 0 | 0 | 0 | 0 | 0 | 0 | 0 | 0 | 0 |
| Corneal Opacity (area) (0-4) | A | OD | 1 | 1 | 1 | 1 | 1 | 1 | 1 | 1 | 1 | 1 | 1 | 1 | 1 |
|  |  | OS | 0 | 0 | 0 | 0 | 0 | 0 | 0 | 0 | 0 | 0 | 0 | 0 | 0 |
|  | B | OD | 0 | 0 | 0 | 0 | 0 | 0 | 0 | 0 | 0 | 0 | 0 | 0 | 0 |
|  |  | OS | 0 | 0 | 0 | 0 | 0 | 0 | 0 | 0 | 0 | 0 | 0 | 0 | 0 |
| Corneal Vascularization (0-2) | A | OD | 0 | 0 | 0 | 0 | 0 | 0 | 0 | 0 | 0 | 0 | 0 | 0 | 0 |
|  |  | OS | 0 | 0 | 0 | 0 | 0 | 0 | 0 | 0 | 0 | 0 | 0 | 0 | 0 |
|  | B | OD | 0 | 0 | 0 | 0 | 0 | 0 | 0 | 0 | 0 | 0 | 0 | 0 | 0 |
|  |  | OS | 0 | 0 | 0 | 0 | 0 | 0 | 0 | 0 | 0 | 0 | 0 | 0 | 0 |
| AC Flare (0-4) | A | OD | 0 | 0 | 0 | 0 | 0 | 0 | 0 | 0 | 0 | 0 | 0 | 0 | 0 |
|  |  | OS | 0 | 0 | 0 | 0 | 0 | 0 | 0 | 0 | 0 | 0 | 0 | 0 | 0 |
|  | B | OD | 0 | 0 | 0 | 0 | 0 | 0 | 0 | 0 | 0 | 0 | 0 | 0 | 0 |
|  |  | OS | 0 | 0 | 0 | 0 | 0 | 0 | 0 | 0 | 0 | 0 | 0 | 0 | 0 |
| Total # of AC cells viewed in volume (field) of the slit beam (0-4) | A | OD | 0 | 0 | 0 | 0 | 0 | 0 | 0 | 0 | 0 | 0 | 0 | 0 | 0 |
|  |  | OS | 0 | 0 | 0 | 0 | 0 | 0 | 0 | 0 | 0 | 0 | 0 | 0 | 0 |
|  | B | OD | 0 | 0 | 0 | 0 | 0 | 0 | 0 | 0 | 0 | 0 | 0 | 0 | 0 |
|  |  | OS | 0 | 0 | 0 | 0 | 0 | 0 | 0 | 0 | 0 | 0 | 0 | 0 | 0 |
| Iris Involvement (0-4) | A | OD | 0 | 0 | 0 | 0 | 0 | 0 | 0 | 0 | 0 | 0 | 0 | 0 | 0 |
|  |  | OS | 0 | 0 | 0 | 0 | 0 | 0 | 0 | 0 | 0 | 0 | 0 | 0 | 0 |
|  | B | OD | 0 | 0 | 0 | 0 | 0 | 0 | 0 | 0 | 0 | 0 | 0 | 0 | 0 |
|  |  | OS | 0 | 0 | 0 | 0 | 0 | 0 | 0 | 0 | 0 | 0 | 0 | 0 | 0 |
| Anterior Vitreous Cell (0-4) | A | OD | 0 | 0 | 0 | 0 | 0 | 0 | 0 | 0 | 0 | 0 | 0 | 0 | 0 |
|  |  | OS | 0 | 0 | 0 | 0 | 0 | 0 | 0 | 0 | 0 | 0 | 0 | 0 | 0 |
|  | B | OD | 0 | 0 | 0 | 0 | 0 | 0 | 0 | 0 | 0 | 0 | 0 | 0 | 0 |
|  |  | OS | 0 | 0 | 0 | 0 | 0 | 0 | 0 | 0 | 0 | 0 | 0 | 0 | 0 |

OD= Right Eye, injected with PBS vehicle control

OS= Left Eye, injected with pIC-activated GFP-labeled MSC

Supplemental Table 3 Continued: Summary of SPOTS over time in horses following pIC-activated GFP-labeled MSC injection

|  |  |  | **Injection 1** | |  |  | **Injection 2** | |  |  | **Injection 3** | |  |  |  |
| --- | --- | --- | --- | --- | --- | --- | --- | --- | --- | --- | --- | --- | --- | --- | --- |
| SPOTS Exam | Horse | Eye | Day 0 | Day 1 | Day 3 | Day 7 | Day 14 | Day 15 | Day 17 | Day 21 | Day 28 | Day 29 | Day 31 | Day 35 | Day 42 |
| Fluorescein Staining (severity) (0-4) | A | OD | 0 | 0 | 0 | 0 | 0 | 1 | 0 | 0 | 0 | 0 | 0 | 0 | 0 |
|  |  | OS | 0 | 0 | 0 | 0 | 0 | 1 | 0 | 0 | 0 | 0 | 0 | 0 | 0 |
|  | B | OD | 0 | 0 | 0 | 0 | 0 | 0 | 0 | 0 | 0 | 0 | 0 | 0 | 0 |
|  |  | OS | 0 | 0 | 0 | 0 | 0 | 0 | 0 | 0 | 0 | 0 | 0 | 0 | 0 |
| Fluorescein Staining (area) (0-4) | A | OD | 0 | 0 | 0 | 0 | 0 | 1 | 0 | 0 | 0 | 0 | 0 | 0 | 0 |
|  |  | OS | 0 | 0 | 0 | 0 | 0 | 1 | 0 | 0 | 0 | 0 | 0 | 0 | 0 |
|  | B | OD | 0 | 0 | 0 | 0 | 0 | 0 | 0 | 0 | 0 | 0 | 0 | 0 | 0 |
|  |  | OS | 0 | 0 | 0 | 0 | 0 | 0 | 0 | 0 | 0 | 0 | 0 | 0 | 0 |
| Lens Opacity (0-1) | A | OD | 1 | 1 | 1 | 1 | 1 | 1 | 1 | 1 | 1 | 1 | 1 | 1 | 1 |
|  |  | OS | 1 | 1 | 1 | 1 | 1 | 1 | 1 | 1 | 1 | 1 | 1 | 1 | 1 |
|  | B | OD | 0 | 0 | 0 | 0 | 0 | 0 | 0 | 0 | 0 | 0 | 0 | 0 | 0 |
|  |  | OS | 0 | 0 | 0 | 0 | 0 | 0 | 0 | 0 | 0 | 0 | 0 | 0 | 0 |
| Vitreous Haze (0-4) | A | OD | 0 | 0 | 0 | 0 | 0 | 0 | 0 | 0 | 0 | 0 | 0 | 0 | 0 |
|  |  | OS | 0 | 0 | 0 | 0 | 0 | 0 | 0 | 0 | 0 | 0 | 0 | 0 | 0 |
|  | B | OD | 0 | 0 | 0 | 0 | 0 | 0 | 0 | 0 | 0 | 0 | 0 | 0 | 0 |
|  |  | OS | 0 | 0 | 0 | 0 | 0 | 0 | 0 | 0 | 0 | 0 | 0 | 0 | 0 |
| Degraded Fundus View (0-3) | A | OD | 0 | 0 | 0 | 0 | 0 | 0 | 0 | 0 | 0 | 0 | 0 | 0 | 0 |
|  |  | OS | 0 | 0 | 0 | 0 | 0 | 0 | 0 | 0 | 0 | 0 | 0 | 0 | 0 |
|  | B | OD | 0 | 0 | 0 | 0 | 0 | 0 | 0 | 0 | 0 | 0 | 0 | 0 | 0 |
|  |  | OS | 0 | 0 | 0 | 0 | 0 | 0 | 0 | 0 | 0 | 0 | 0 | 0 | 0 |
| Retinal Perivascular Sheathing (0-3) | A | OD | 0 | 0 | 0 | 0 | 0 | 0 | 0 | 0 | 0 | 0 | 0 | 0 | 0 |
|  |  | OS | 0 | 0 | 0 | 0 | 0 | 0 | 0 | 0 | 0 | 0 | 0 | 0 | 0 |
|  | B | OD | 0 | 0 | 0 | 0 | 0 | 0 | 0 | 0 | 0 | 0 | 0 | 0 | 0 |
|  |  | OS | 0 | 0 | 0 | 0 | 0 | 0 | 0 | 0 | 0 | 0 | 0 | 0 | 0 |
| Intraocular pressure (mmHg) | A | OD | 20 | 17 | 19 | 19 | 19 | 17 | 19 | 16 | 20 | 15 | 17 | 18 | 15 |
|  |  | OS | 20 | 22 | 20 | 17 | 18 | 15 | 16 | 15 | 16 | 22 | 19 | 18 | 15 |
|  | B | OD | 22 | 15 | 17 | 18 | 17 | 15 | 15 | 18 | 14 | 12 | 18 | 14 | 14 |
|  |  | OS | 15 | 14 | 15 | 19 | 17 | 17 | 14 | 17 | 15 | 14 | 17 | 14 | 14 |

OD= Right Eye, injected with PBS vehicle control

OS= Left Eye, injected with pIC-activated GFP-labeled MSC

Supplemental Table 2: Summary of Complete Blood Counts analytes over time in horses following pIC-activated GFP-labeled MSC injection

|  |  | **Injection 1** | |  |  | **Injection 2** | |  |  | **Injection 3** | |  |  |  |
| --- | --- | --- | --- | --- | --- | --- | --- | --- | --- | --- | --- | --- | --- | --- |
| **Erythrocyte Parameters** | Horse | Day 0 | Day 3 | | Day 7 | Day 14 | Day 17 | | Day 21 | Day 28 | Day 31 | | Day 35 | Day 42 |
| RBC (M/uL) | A | 5.23 | 6.21 | | 6.23 | 5.67 | 5.61 | | 6.23 | 6.08 | 6.29 | | 6.51 | 6.96 |
| Ref (6.2-10.2) | B | 8.38 | 7.32 | | 8.15 | 8.98 | 6.86 | | 8.01 | 7.63 | 8.44 | | 9 | 8.86 |
| Hemoglobin (gm/dl) | A | 9.6 | 11.4 | | 11.5 | 10.4 | 10.5 | | 11.4 | 11.2 | 11.5 | | 11.7 | 12.8 |
| Ref (11.2 -17.2) | B | 14.3 | 12.7 | | 14 | 15.5 | 11.7 | | 13.8 | 13.2 | 14.7 | | 15.5 | 15.3 |
| Hematocrit (%) | A | 26.9 | 32.1 | | 32.7 | 29.1 | 28.9 | | 32.2 | 31.1 | 32.5 | | 33.4 | 35.8 |
| Ref (30-46) | B | 39.2 | 34.1 | | 38.3 | 42 | 31.8 | | 37 | 35.7 | 39.6 | | 42.2 | 41.6 |
| **Leukocyte Parameters** |  |  |  | |  |  |  | |  |  |  | |  |  |
| WBC ( /uL ) | A | 4,080 | 4,630 | | 4,890 | 5,650 | 4,430 | | 4,950 | 4,630 | 4,800 | | 4,840 | 5,630 |
| Ref (5,000-11,600) | B | 7,990 | 6,440 | | 6,400 | 8,070 | 5,510 | | 5,920 | 6,080 | 6,210 | | 6,870 | 8,210 |
| Neutrophils ( /uL ) | A | 2,676 | 2,982 | | 3,105 | 3,899 | 3,008 | | 3,510 | 3,093 | 3,149 | | 3,630 | 3,913 |
| Ref (2,600-6,800) | B | 4,514 | 3,123 | | 3,392 | 3,970 | 2,733 | | 2,581 | 2,937 | 3,080 | | 3,696 | 4,294 |
| Lymphocytes ( /uL ) | A | 1,159 | 1,324 | | 1,418 | 1,526 | 1,156 | | 1,129 | 1,269 | 1,320 | | 1,065 | 1,396 |
| Ref (1,600-5,800) | B | 3,132 | 2,975 | | 2,675 | 3,688 | 2,579 | | 3,126 | 2,876 | 2,838 | | 2,817 | 3,571 |
| Monocytes ( /uL ) | A | 135 | 157 | | 176 | 226 | 137 | | 134 | 111 | 173 | | 73 | 186 |
| Ref (0-500) | B | 296 | 270 | | 275 | 331 | 160 | | 172 | 207 | 186 | | 275 | 271 |
| Eosinophils ( /uL ) | A | 98 | 139 | | 176 | 0 | 115 | | 149 | 144 | 130 | | 48 | 118 |
| Ref (0-200) | B | 24 | 58 | | 38 | 40 | 22 | | 24 | 36 | 62 | | 48 | 41 |
| Basophils ( /uL ) | A | 12 | 32 | | 15 | 0 | 13 | | 30 | 19 | 34 | | 24 | 17 |
| Ref (0-100) | B | 16 | 13 | | 19 | 32 | 22 | | 12 | 18 | 43 | | 34 | 25 |
| **Other Parameters** |  |  |  | |  |  |  | |  |  |  | |  |  |
| Platelets (/uL) | A | 148,000 | 152,000 | | 160,000 | 198,000 | 194,000 | | 188,000 | 171,000 | 179,000 | | 195,000 | 207,000 |
| Ref (100,000-225,000) | B | 140,000 | 114,000 | | 109,000 | 112,000 | 113,000 | | 8,000 | 110,000 | 82,000 | | 104,000 | 128,000 |
| MPV (fl) | A | 6.6 | 6.5 | | 6.6 | 6.3 | 6.8 | | 6.8 | 6.2 | 6.4 | | 6.8 | 7.3 |
| Ref (5.4-9.3) | B | 7.5 | 6.4 | | 6.6 | 6.5 | 6.8 | | 11 | 6.1 | 6.9 | | 6.4 | 7.8 |
| Plasma Protein (gm/dL) | A | 7 | 6.8 | | 7.2 | 6.9 | 7 | | 7 | 7 | 6.8 | | 6.6 | 7 |
| Ref (5.8-8.7) | B | 7 | 6.6 | | 6.5 | 7 | 6.2 | | 6.1 | 6.2 | 6.6 | | 6.6 | 6.4 |
| Plasma Fibrinogen (mg/dL) | A | 200 | 200 | | 300 | 200 | 200 | | 300 | 200 | 200 | | 100 | 200 |
| Ref (100-400) | B | 200 | 200 | | 200 | 200 | 200 | | 100 | 200 | 200 | | 100 | 200 |

Values highlighted in yellow for Horse A and green for Horse B are outside laboratory reference range
