# Supplementary material for: Preliminary evaluation of safety and migration of immune activated mesenchymal stromal cells administered by subconjunctival injection for equine recurrent uveitis
Source: Front Vet Sci. 2023 Dec 14;10:1293199. doi: 10.3389/fvets.2023.1293199 (PMC10757620; doi:10.3389/fvets.2023.1293199)
Supplement: Supplementary file 4 [file Data_Sheet_1.docx]

**Supplemental Material 1: Tissues collected and examined microscopically**

Brainstem

Cerebellum

Cerebrum

Thalamus

C1, T17, L1 spinal cord segments

Heart

Kidney

Liver

Lungs

Spleen

Thyroid gland

Adrenal gland

Large intestine

Small intestine

**Supplemental Material 2: Tissues collected and examined with IVIS Spectrum**

Eyes: Exenerated (containing conjunctiva and lids), slit dorsal lid margin at 12 o’clock to reflect and allow for imaging of dorsal bulbar conjunctiva and dorsal eyelid

Heart- Left Atria, Right Atria, Left Ventricle, Right Ventricle, Septum

Kidney-Left and Right

Liver

Lung- Left cranial, left caudal, right cranial, right caudal

Spleen

Pancreas

Adrenal Gland- Left and Right

Mandibular salivary gland

Lymph Node- Submandibular, Pharyngeal, Tracheobronchial, Mesenteric

Sciatic Nerve

Skeletal Muscle-hind limb

Bladder

Tongue

Esophagus

Stomach

Small intestine- Duodenum, Jejunum, Ileum

Large Intestine- Left Dorsal Colon, Left Ventral Colon, Right Dorsal Colon, Right Ventral Colon, Cecum, Transverse Colon
